# Sandwich-type architecture film based on WS_2_ and ultrafast self-expanded and reduced graphene oxide in a Li-ion battery

**DOI:** 10.3389/fchem.2022.1102207

**Published:** 2023-01-16

**Authors:** Karolina Wenelska, Tomasz Kędzierski, Damian Bęben, Ewa Mijowska

**Affiliations:** ^1^ Department of Nanomaterials Physicochemistry, Szczecin Faculty of Chemical Technology and Engineering, West Pomeranian University of Technology, Szczecin, Poland; ^2^ Nanores Sp. z o.o. Sp.k, Wroclaw, Poland; ^3^ Institute of Low Temperature and Structure Research, Polish Academy of Sciences in Wroclaw, Wroclaw, Poland

**Keywords:** tungsten disulfide (WS_2_), graphene oxide, batterie, composites, film

## Abstract

Since its discovery, graphene has been widely considered a great material that has advanced the Li-ion battery field and allowed development in its performance. However, most current graphene-related research is focused on graphene-based composites as electrode materials, highlighting the role of graphene in composite materials. Herein, we focused on a three-dimensional composite film with unique sandwich-type architecture based on ultrafast self-expanded and reduced graphene oxide (userGO) and exfoliated WS_2_. This strategy allows non-active agents [e.g., carbon black and poly (vinylidene fluoride)] free electrodes in LIBs in the form of a film. The ultra-quick exothermal nature of the USER reaction allows the rapid release of internally generated gases to create highly porous channels inside the film. Hence, the improved Li-ion transport in the LIBs boosted the electrochemical performance of both film components (ex-WS_2_ and reduced graphene), resulting in a high specific capacity of 762 mAh/g at .05 A/g and high Coulombic efficiency (101%) after 1,000 cycles. Overall, userGO showed the highest capacity at a low current, and ex-WS_2_ provided a higher reversible capacity. These results showed that the expanded graphene layer is an excellent shield for ex-WS_2_ to protect against pulverization, promoting both stability and capacity.

## 1 Introduction

With ever-increasing global energy demands and allied to efforts to reduce the use of fossil fuels and eliminate air pollution, it is essential to provide efficient, cost-effective, and environmentally friendly energy storage devices like lithium-ion batteries, supercapacitors or fuel cells (Sengupta and Kundu, 2020). Lithium-ion batteries (LIBs) are predominant energy storage systems used in portable devices, electrical vehicles, and other applications because of their high energy density and long cycling performance (Li et al., 2022). Many researchers have focused on the development of various electrochemically active materials, including silicon, metal oxides, and transition metal disulfides and their composite materials as anode materials for LIBs (Bates et al., 2000; Ali et al., 2022). In the last decade, transition-metal dichalcogenides (TMDs) have received attention as electrode materials for Li-ion batteries owing to their high electronic conductivity, superior specific capacity, marvelous structural stability, and environment benignity (Shin et al., 2021). The interlayer structure of TMDs comprises three stacked atom layers (X–M–X) held together by van der Waals forces between the transition metal (M) and the chalcogenide anions (X), allowing easy Li^+^ ion insertion/de-insertion (Chen H et al., 2020). In particular, the WS_2_ nanomaterials have attracted attention and have been utilized as solid lubricant photosensitive films and super shock absorbers. Additionally, WS_2_ has a superior volumetric energy density (3,248 mAh cm^−3^) due to its large density (7.5 g cm^−3^); these advantages make WS_2_ favorable for LIBs. However, the electron conductivity of tungsten disulfide is too low, which could lead to rapid capacity fading and poor rate performance when used as the anode material (Feng et al., 2007; Kartick et al., 2014). Therefore, conductive additives based on carbon materials such as graphene, reduced graphene oxide, single-/multi-walled C nanotubes, and pyrolytic C, which enhance the electrochemical performance without side reactions involving Li-ion-batteries, have been used to enrich WS_2_-based electrodes. For example, Li et al. (2016) described a nanocomposite of WS_2_ and Super P carbon black (WS_2_/C) as an anode material for Na-ion batteries (NIBs) and LIBs. The resulting anode exhibited a significantly enhanced electrochemical performance compared to that of a pristine WS_2_ anode, which could be attributed to the high conductivity of Super P carbon black in cycling. Ren et al. (2018) successfully fabricated the foam structure of WS_2_/single-wall carbon nanotube nanocomposites, which delivered a reversible capacity (∼688 mAh g^−1^) for 1,000 cycles at 0.1 A g^−1^. Many researchers have focused on graphene, which is a perfect compound material with advanced electrochemical characteristics and mechanical strength. The similar 2D structures of graphene and layered WS_2_ maximize their geometrical compatibility and the possibility of stronger component interactions, leading to favorable outcomes. Zhou et al. (2016a) presented a one-pot method for the synthesis of a WS_2_/reduced graphene oxide (rGO) composite, which dramatically improved battery performance. The WS_2_/rGO anode showed a stable discharge capacity of 431.2 mAh/g at a current density of 0.1 A/g after 100 cycles, which could be improved.

Herein, we propose a conceptually new and facile method to fabricate a novel three-dimensional composite with unique sandwich-type architecture based on exfoliated WS_2_ and ultrafast self-expanded and reduced graphene oxide (userGO). This molecular nanostructure served as a non-active agent-free electrode in LIBs in the form of a film to promote its electrochemical response compared to the composite based on exfoliated WS_2_ with conventionally thermally-reduced GO in an oven. The integration of WS_2_ and userGO into a composite was accomplished using the layer-by-layer vacuum filtration technique (Cheng et al., 2019) with precise concentrations of the respective components followed by the ultrafast and self-expanded reduction (USER) reaction occurring in the glovebox under an inert gas atmosphere at 350°C (Chen Y. W et al., 2020). The highly exothermic character of the USER reaction immediately starts from the touchpoint and rapidly spreads across the whole film, changing its color from grey to black. The ultra-quick nature of the USER reaction allows it to propagate easily on the WS_2_/userGO film from a single point of contact. These internally generated gases were rapidly released from the WS_2_/GO film to create highly porous channels inside the film to increase the film thickness and expansion. The modified WS_2_/userGO film showed superior electrochemical performance owing to the effective exothermal USER reaction method to generate increased distances between sandwich structures to improve ion transport in LIBs.

## 2 Experimental methods

### 2.1 Synthesis of graphene oxide (GO)

In the typical procedure, graphene oxide (GO) was produced from pure graphite powder using a modified Hummers method (Marcano et al., 2010). In this method, concentrated sulfuric acid and orthophosphoric acid (120:15 mL) were added to a mixture of KMnO_4_ (6 g) and graphite (1 g). This mixture was stirred for 24 h at 50°C until the solution became dark green. To eliminate excess KMnO_4_, 150 ml of hydrogen peroxide (H_2_O_2_) was dropped slowly, stirred for 10 min, and filtered using a polycarbonate membrane. The solid product was washed with water, 30% HCl, and ethanol twice before vacuum drying for 12 h.

### 2.2 Exfoliation of WS_2_ (ex-WS_2_)

Few-layered WS_2_ nanosheets were obtained by ultrasound-assisted liquid exfoliation using N-methylpyrrolidone (NMP) as a solvent. Briefly, bulk WS_2_ (1 g) and NMP (100 mL) were dispersed by sonication and stirred for 5 h at 35°C. The mixture was then centrifuged and the supernatant replaced with fresh NMP. The obtained product was centrifuged, washed with ethanol several times, and dried in a vacuum at 80°C.

### 2.3 WS_2_/userGO film preparation

WS_2_/GO films were synthesized using a microfiltration set. In the typical procedure, GO (15 mg) was dispersed in distilled water (500 ml) and 15 mg of WS_2_ was dispersed in ethanol (300 mL) *via* sonication. Three ex-WS_2_:GO dispersions with 1:1, 1:2, and 2:1 weight ratios were obtained, respectively. Next, the mixture was poured into the filtration set (You et al., 2013). The obtained ex-WS_2_/GO films were dried at 25°C overnight. The freestanding films were placed in the glovebox in an argon atmosphere. When the respective ex-WS_2_/GO film was initially put into contact with a 350°C hotplate, the USER reaction happened immediately from the touchpoint and quickly spread across the whole film. During this reaction, the color of the film changed immediately from grey to black. Expanded thickness, smoke, and sparks from the films were also observed. The resulting samples were named WS_2_/userGO1 (1:1), WS_2_/userGO2 (1:2), and WS_2_/userGO3 (2:1).

### 2.4 Characterization

TEM images were taken directly on sample-drop-casted Cu grids with a carbon film using a TECNAI F30 microscope at 200 kV. The surface topologies of the composite components were determined by AFM imaging on Si wafers using a Nanoscope V Multimode 8 instrument. Postprocessing of the AFM data was performed using the included software. The samples for TEM and AFM were made by dispersing the samples in isopropyl alcohol and in a mixture of isopropyl alcohol and water (20:1), respectively, followed by sonication for 30 min and drying for 24 h. To determine the morphology of the obtained samples, scanning electron microscopy (SEM) was performed on an SEM/Ga-FIB FEI Helios NanoLabTM 600i dual beam microscope. X-ray diffraction (X-ray diffractometer Philips X’Pert PRO, PANalytical B.V., Kα 1 = 1.54056 Å) was used to investigate the film structures. Raman spectroscopy was applied (InViaRenishaw) for the characterization of the samples (785 nm laser).

### 2.5 Electrochemical measurements

Thin electrodes (∼12 mm) composed only of active material (WS_2_/userGO-based films) in three different ratios were cut out. No binding/conductive agents nor any substrate were required. However, reference ex-WS_2_ electrodes were prepared in the form of tablets with the addition of carbon black (CB) and poly (vinylidene fluoride) (PVDF) in a 4.5:4.5:1 ratio, respectively. Before assembling the half-cells, the electrodes were dried in a vacuum at 100°C overnight. The cells were then assembled in a half-cell configuration. Metallic lithium acted as reference and counter electrode (cathode) and the prepared films were used as working electrodes (anode). The coin cells were assembled inside a glovebox filled with argon (MBraun UNILab PLUS SP). Before the electrochemical measurements, the cells were attached to the potentiometer (Biologic VMP3) for 8 h to apply the open-circuit voltage (OCV) technique. Galvanostatic charge/discharge cycling was performed on 0–3 V vs. Li/Li^+^. Cyclic voltammetry (CV) was executed at a scan rate of 0.1 mV/s at room temperature. The impedance (EIS) was then tested in the frequency range from 100 kHz to 10 mHz. Galvanostatic cycling with potential limitation (GCPL) was used to determine the long-term stability (1,000 cycles at 0.1 A/g) and specific capacities of the cells at current densities of 0.05, 0.1, 0.2, 0.4, 0.6, 1, 2, 5 and 10 A/g and again at 0.05 A/g. The specific capacity was calculated based on the mass loading of the active materials.

## 3 Results and discussion

**SCHEME 1:**
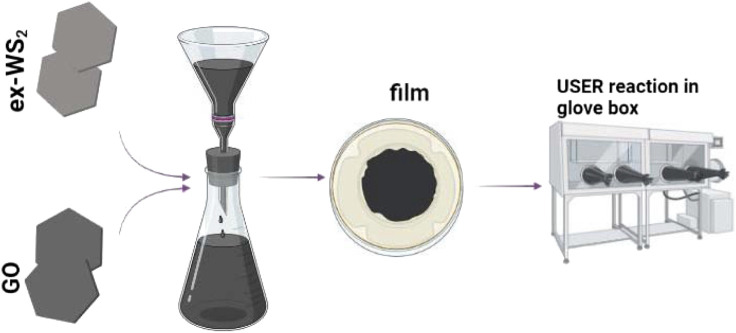
Illustrations of the synthesis process.

**FIGURE 1:**
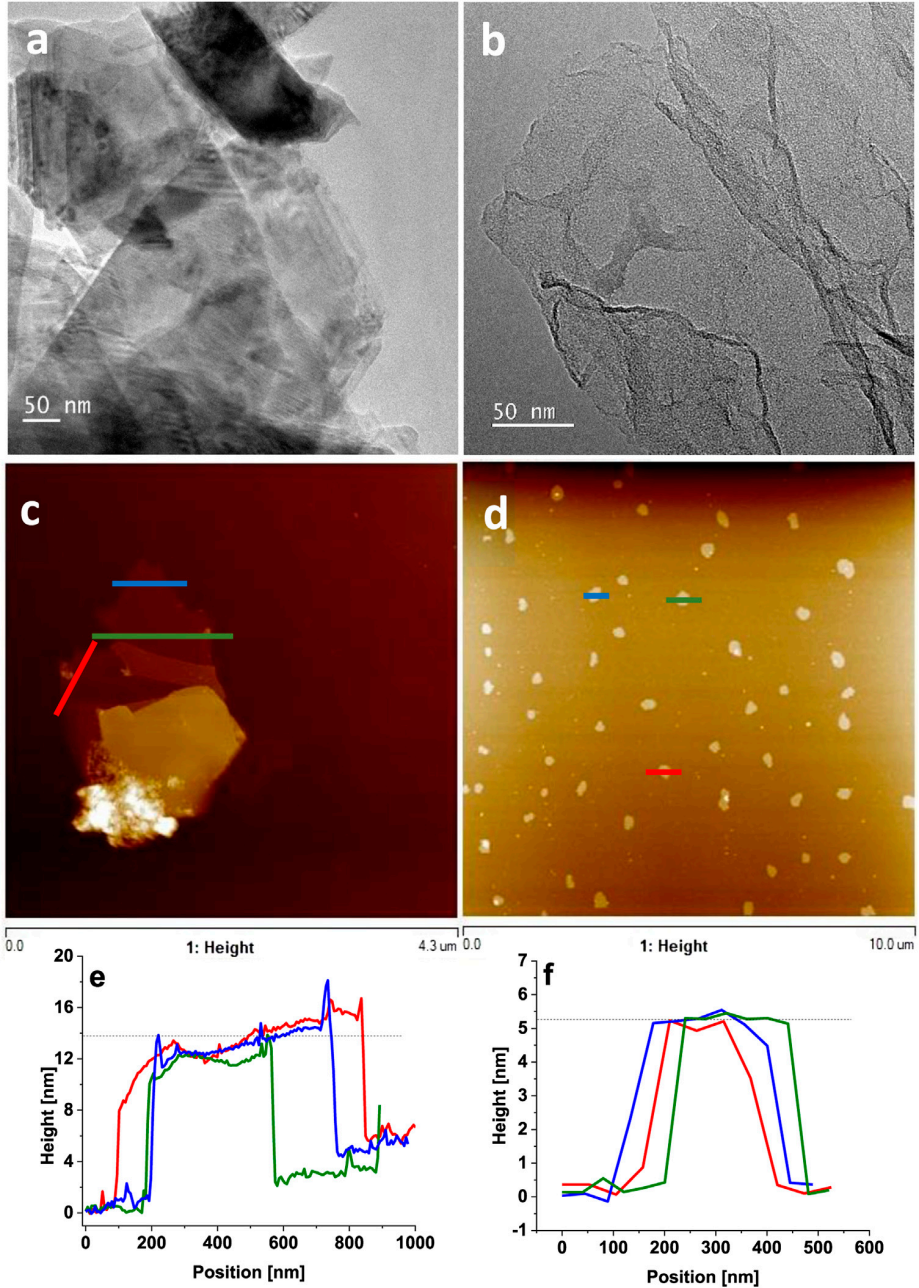
TEM and AFM images of **(A)** WS_2_, **(B)** GO, **(C)** WS_2_, and **(D)** GO and height profiles of **(E)** WS_2_ and **(F)** GO.

Successful preparation of few-layered WS_2_ and GO samples as shown in Scheme 1 were confirmed by TEM (Figures 1A, B) and AFM (Figures 1C, D). The TEM images of both film components exhibited flake-like structures typical for 2D materials. Moreover, the analysis indicated that the samples contained no impurities. The number of WS_2_ and GO layers was determined through high-profile analysis with AFM (Figures 1E, F). The flakes were typically 14 nm in height for WS_2_ and 5 nm for GO, which corresponded to approximately 20 and 7 layers of WS_2_ (Xu et al., 2017) and GO (You et al., 2013), respectively.

**FIGURE 2:**
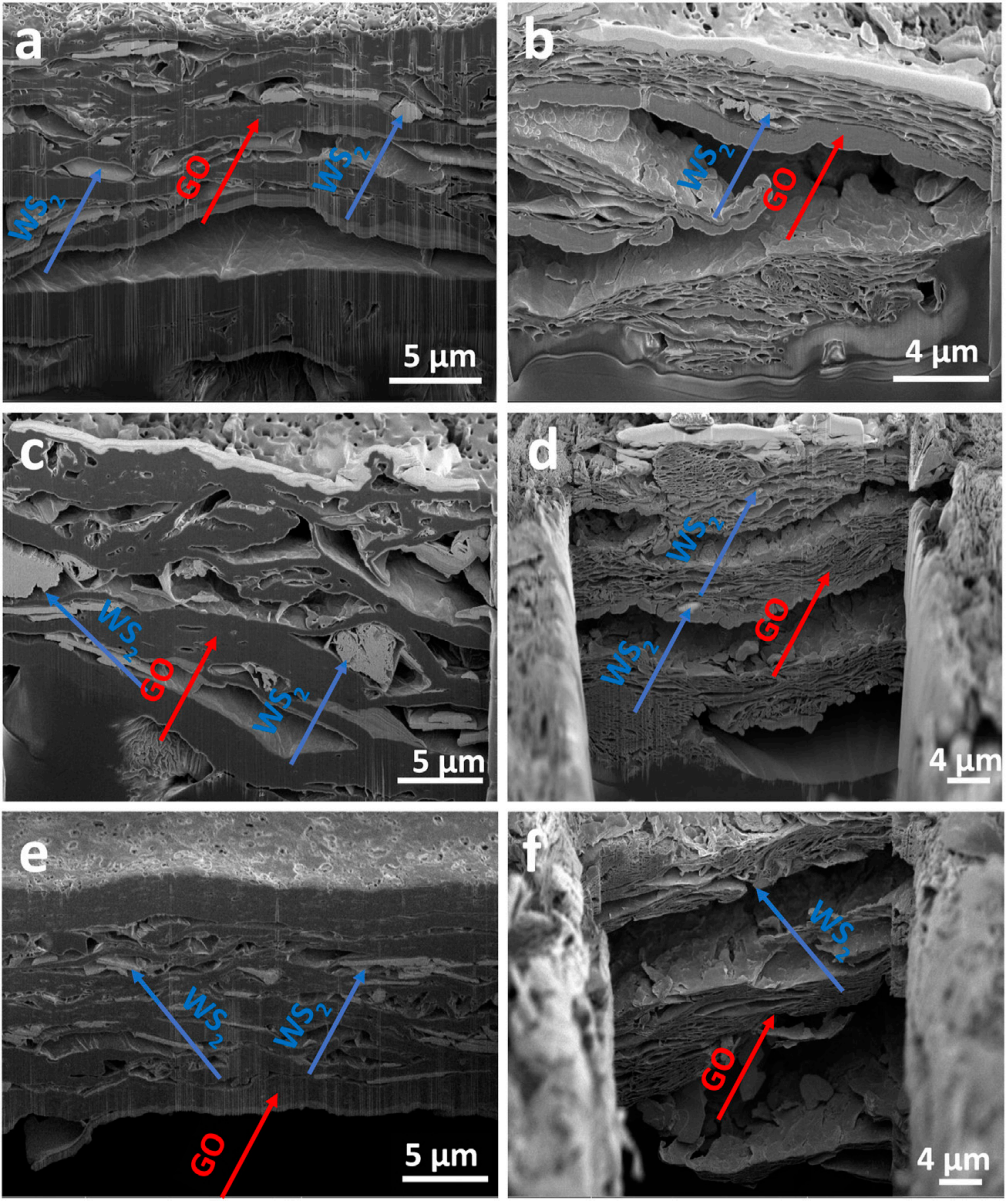
SEM images of WS_2_/userGO1 **(A)** before and **(B)** after the USER reaction; WS_2_/userGO2 **(C)** before and **(D)** after the USER reaction, and WS_2_/userGO3 **(E)** before and **(F)** after the USER reaction.

The morphologies of the cross-sections of the obtained films before and after the USER reaction with different ex-WS_2_ ratios were studied by SEM, as shown in Figures 2A–F. Before the USER reaction, the films were characterized by condensed and compact phases of GO (dark gray areas) and ex-WS_2_ (light gray areas). The ex-WS_2_ sheets were located between GO layers. After the USER reaction, the structure expanded. The release of the gases and internal expansion of the film structures created cavities in the GO structure. Additionally, larger distances were observed between the layers in both ex-WS_2_ and GO. Therefore, a more efficient lithiation/delithiation process was expected.

**FIGURE 3:**
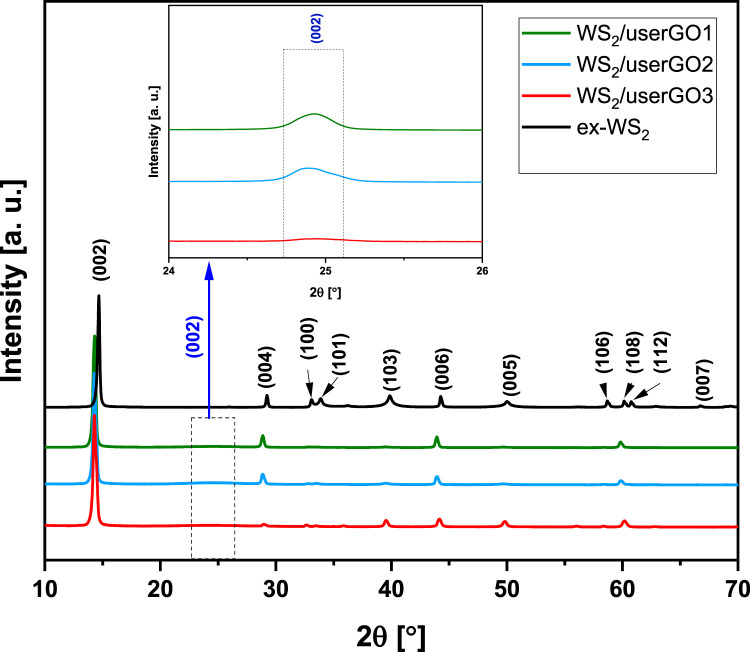
XRD pattern of WS_2_/userGO1, WS_2_/userGO2, WS_2_/userGO3 film, and ex-WS_2_.

The crystal structure and phase purity of the synthesized molecular hybrids were verified and compared to exfoliated WS_2_ by XRD (Figure 3). The diffraction patterns of the three prepared samples were similar to those for ex-WS_2_. The diffraction peaks of the films with different components ratio located at 14.7°, 29.2°, 33.2°, 34°, 39.9°, 44.3°, 50°, 58.8°, 60.2°, 61° were assigned to the (002), (004), (100), (101), (103), (006), (005), (106), (108), and (112) planes of WS_2_, respectively (ICDD card no. 04-004-4224). Moreover, an intense and sharp (002) peak observed in the XRD pattern indicated that the WS_2_/userGO films were stacked together with a highly ordered packing (Vattikuti et al., 2016). Additionally, a clear shift of WS_2_ peaks to lower 2θ angles was observed in all WS_2_/userGO films compared to ex-WS_2_, indicating increased d-spacing in the ex-WS_2_ after the USER reaction. The USER reaction removes most of the oxygenated functionalities from the surface of the graphene oxide sheet, reducing the structure to graphene, which was detected in diffraction angles at 12°, 23°, 42° corresponding to (001), (002), and (100) planes (Supplementary Figure S1).

**FIGURE 4:**
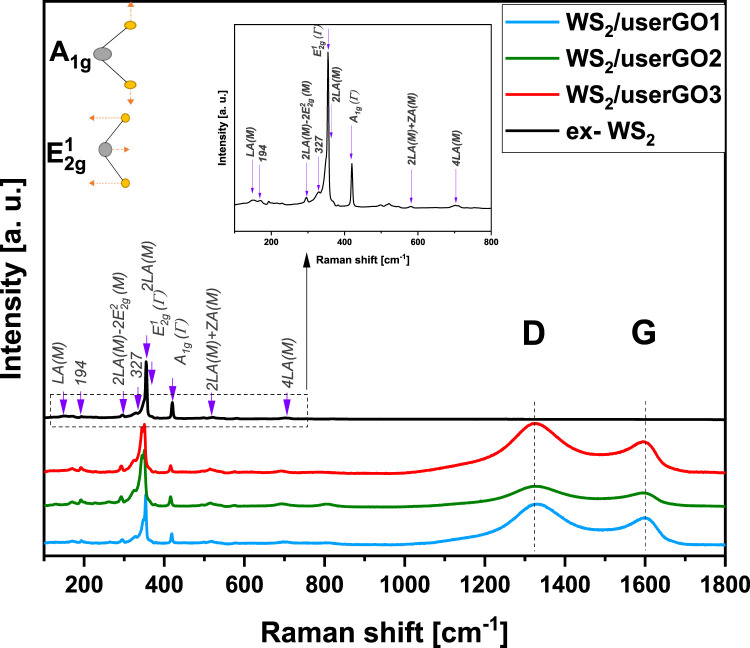
Raman spectroscopy of WS_2_/userGO1, WS_2_/userGO2, WS_2_/userGO3 film, and ex- WS_2._

The Raman spectra for ex-WS_2_ and WS_2_/userGO films are shown in Figure 4. The Raman spectra for the samples were similar in range, from 150 to 800 cm^−1^. Two Raman scattering peaks at 417 cm^−1^ and 349 cm^−1^ were also detected. These two peaks [A_1g_(Γ) and E^1^_2g_ (Γ), respectively] are usually observed in the back-scattering configuration. In a backscattering geometry, these spectra include first-order modes at the Brillouin zone center [A_1g_(Γ) and E^1^_2g_ (Γ)] (Berkdemir et al., 2013). The remaining Raman bands were correlated with the second-order modes at the M point and were a combination or difference of bands coupled with the longitudinal acoustic mode LA(M). The LA(M) mode indicates the in-plane collective movements of the atoms in the lattice. The WS_2_/userGO-based films displayed several Raman modes characteristic of WS_2_. However, the relative intensities of the first and second-order modes of the films differed from those of the ex-WS_2_. The decreased band intensity may be associated with more efficient exfoliation, which might have occurred in the ex-WS_2_ layered structure during the USER reaction. The Raman spectra of the films showed two additional peaks characteristic of carbon materials. The D peak arising from the doubly resonant disorder-induced mode (∼1,323 cm^−1^) due to the stretching of the C–C bond and the G peak, a double phonon mode occurring due to the first-order scattering of the of the sp (Li et al., 2022) C atoms at the Brillouin zone center (∼1,600 cm^−1^). The films showed a prominent D peak, indicative of significant structural disorder due to the incorporation of WS_2_ flakes and the occurrence of USER reaction and is related to the sizes of the in-plane sp (Li et al., 2022) domains and defects (Dresselhaus et al., 2005).

**FIGURE 5:**
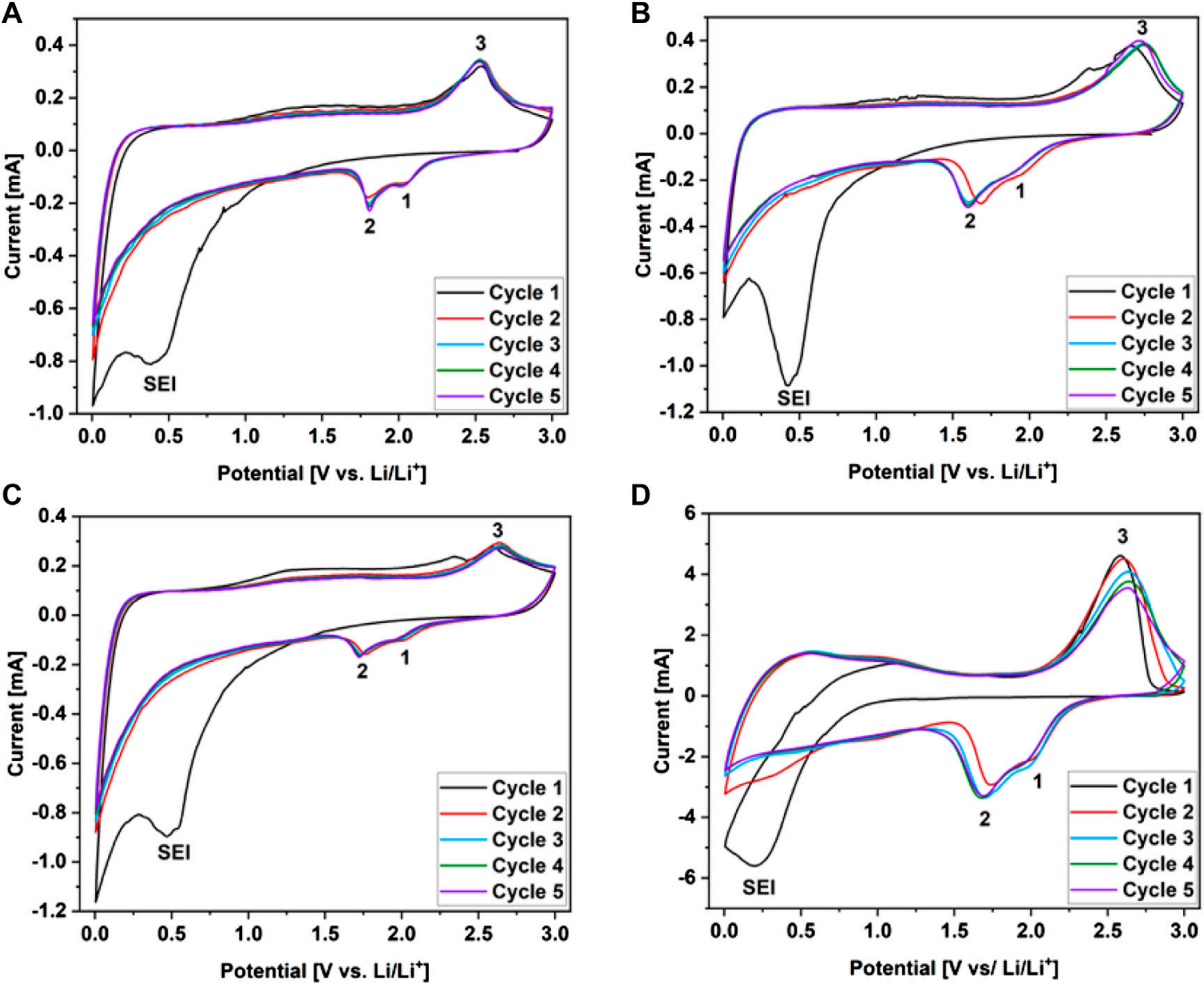
Cyclic voltammograms recorded over 0–3.0 V for **(A)** WS_2_/userGO1, **(B)** WS_2_/userGO2, **(C)** WS_2_/userGO3, and **(D)** ex-WS_2_.

The electrochemical performances of the WS_2_/userGO-based films and reference ex-WS_2_ were investigated using half-cells. In this configuration, metallic lithium acted as reference and counter electrodes, while the prepared materials acted as working electrodes. The five cycles of cyclic voltammetry (CV) of WS_2_/userGO-based films are presented in Figures 5A–C. All these plots showed three redox peaks: 1) at ∼ 2 V and 2) at ∼ 1.8 V, indicating the two-step reduction of W^4+^ to metallic W, as shown in Eq. 1, 2, respectively; 3) a peak at ∼2.5 V, confirming the oxidation of W to W^4+^ and, thus, the reformation of WS_2_ (Eq. 3). These findings verified the reversibility of the redox process in the charge/discharge tests (Shiva et al., 2013). Another signal at ∼0.5 V corresponded to irreversible SEI layer formation. Due to ex-WS_2_ pulverization upon charging/discharging, a higher amount of CB was added to an electrode to improve the stability of the reference ex-WS_2_ electrode (Figure 5D). However, the CV response of this reference film was less stable than that observed in the composite films, as shown by the better overlapping of the curves in the following cycles. The CV tests proved the excellent stability and electrochemical reversibility of the WS_2_/userGO-based films as lithium-ion storage. The CV test results showed that WS_2_/userGO1 had the highest potential for electrochemical energy conversion.

$${WS}_{2}+{xLi}^{-}+e^{-}\to {Li}_{x}{WS}_{2}\;\left(x=3-4\right),$$ (1)

$${Li}_{x}{WS}_{2}+\left(4-x\right)\;{Li}^{+}+\left(4-x\right)\;e^{-}\to 2{Li}_{2}S+W,$$ (2)

$${xLi}_{2}S+W\to xLi+{WS}_{2}.$$ (3)

**FIGURE 6:**
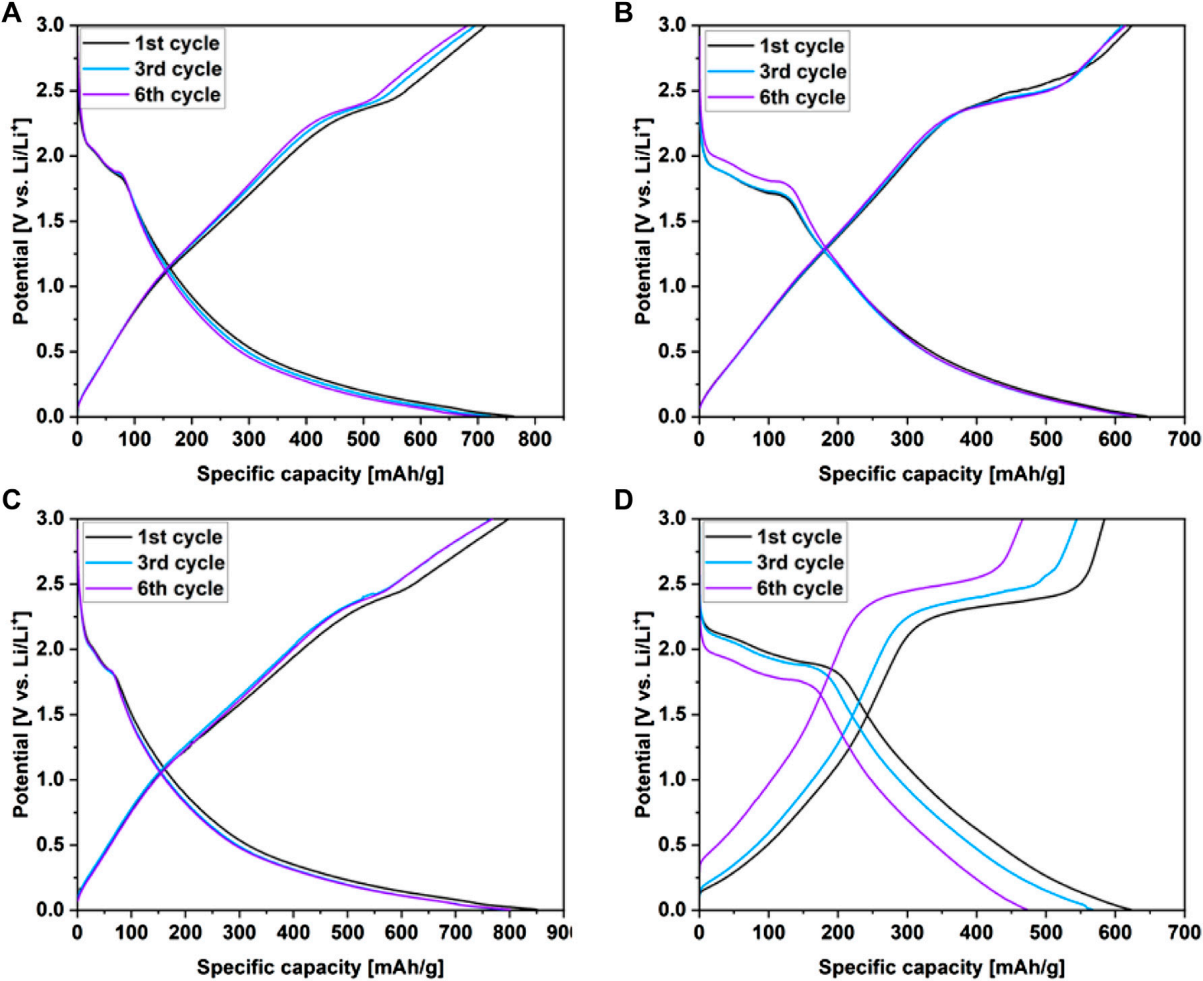
Charge/discharge profiles recorded over .05 A/g for **(A)** WS_2_/userGO1, **(B)** WS_2_/userGO2, **(C)** WS_2_/userGO3, and **(D)** ex-WS_2_.

Figure 6 shows the galvanostatic discharge/charge curves (GCD) of the WS_2_/userGO-based films and reference ex-WS_2_ electrode executed over 0.05 A/g. The initial charge/discharge specific capacities were 714/762, 624/645, 798/851, and 584/622 mAh/g for WS_2_/userGO1, WS_2_/userGO2, WS_2_/userGO3, and ex-WS_2_, respectively. Additionally, the initial specific capacities of all electrodes are shown in Table 1. The highest and lowest capacities were observed for WS_2_/userGO3 and the reference ex-WS_2_, respectively. These results suggested that the unique structure of userGO plays a greater role in lithiation yield compared to ex-WS_2_. This occurred mostly due to the expanded structure of userGO, which allowed a smoother de-/lithiation process. In the case of WS_2_/userGO-based electrodes (Figures 6A–C), the following cycles showed insignificant capacity fading due to the excellent stability of the userGO framework. The films exhibited plateaus at 2 and 1.8 V (in the discharge region), matching the two-step reduction of ex-WS_2_ into metallic tungsten. An oxidation plateau at ∼2.5 V corresponded to the reformation of ex-WS_2_ during charging. These plateaus were most detectable in the pristine ex-WS_2_ electrode (Figure 6D) and this analysis is analogous to the CV measurements. The presence of carbon black in the ex-WS_2_ electrode did not protect the device against a huge capacity drop. However, userGO not only protected the ex-WS_2_ from structure degradation upon charging/discharging but also further increased the capacity than when CB was introduced to the ex-WS_2_ reference electrode.

**TABLE 1:** Initial charge/discharge capacities of electrodes examined at 0.05 A/g.

Working electrode	Charge capacity [mAh/g]	Discharge capacity [mAh/g]
WS_2_/userGO1	**714**	**762**
WS_2_/userGO2	**624**	**645**
WS_2_/userGO3	**798**	**851**
ex-WS_2_	**584**	**622**

**FIGURE 7:**
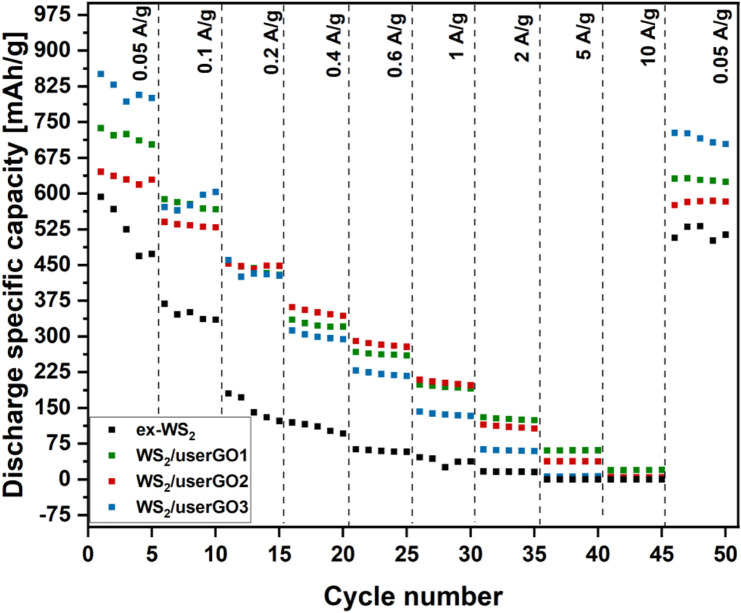
Rate performances of WS_2_/userGO1, WS_2_/userGO2, WS_2_/userGO3, and ex-WS_2_ electrodes recorded at different current densities.

The rate performances of WS_2_/userGO-based films and ex-WS_2_ are depicted in Figure 7. The tests were performed at 0.05, 0.1, 0.2, 0.4, 0.6, 1, 2, 5, and 10 A/g current densities, with reversible capacity at 0.05 A/g. Although WS_2_/userGO1 did not show the highest capacity, it was the most electrochemically durable material; its capacity remained the highest at high current densities among all the samples. Moreover, it showed excellent reversible capacity, keeping 86% of the initial capacity at 0.05 A/g. WS_2_/userGO2 showed decreased capacity in low-to-high performance, although the reverse capacity retained 89% of the initial capacity. WS_2_/userGO3 shows the cyclic performance of WS_2_/userGO3, which showed the highest specific capacity among the tested materials at low current densities but the lowest specific capacity at high current densities. It also maintained the lowest extent in reverse capacity among studied films (85%). Therefore, userGO abundance provided the highest capacity at low current and ex-WS_2_ maintained a higher reversible capacity. Therefore, equal loading of the film components (WS_2_/userGO1) is the most optimal composition for overall electrochemical performance. Additionally, ex-WS_2_ presents the rate performance of the reference ex-WS_2_. Due to irreversible structure changes during the charge/discharge process, the electrochemical performance of ex-WS_2_ worsened significantly. The electrode with the reference ex-WS_2_ was prepared traditionally with other non-active agents. It was physically impossible to prepare a free-standing film using the proposed strategy based only on ex-WS_2._ The ratio of ex-WS_2_ to CB to PVDF was 4.5:4.5:1, respectively. The specific capacity of ex-WS_2_ at 0.05 A/g was almost as high as that of the WS_2_/userGO-based electrodes; however, it deteriorated drastically in the following cycles. Therefore, the presence of carbon buffer in the form of CB was not sufficient to fully protect ex-WS_2_ from pulverization. These results demonstrated that userGO was an excellent shield for ex-WS_2_ boosting both stability and capacity. Moreover, assembling userGO and ex-WS_2_ composites as free-standing films reduced the electrode mass and made it more electrochemically and mechanically durable.

**FIGURE 8:**
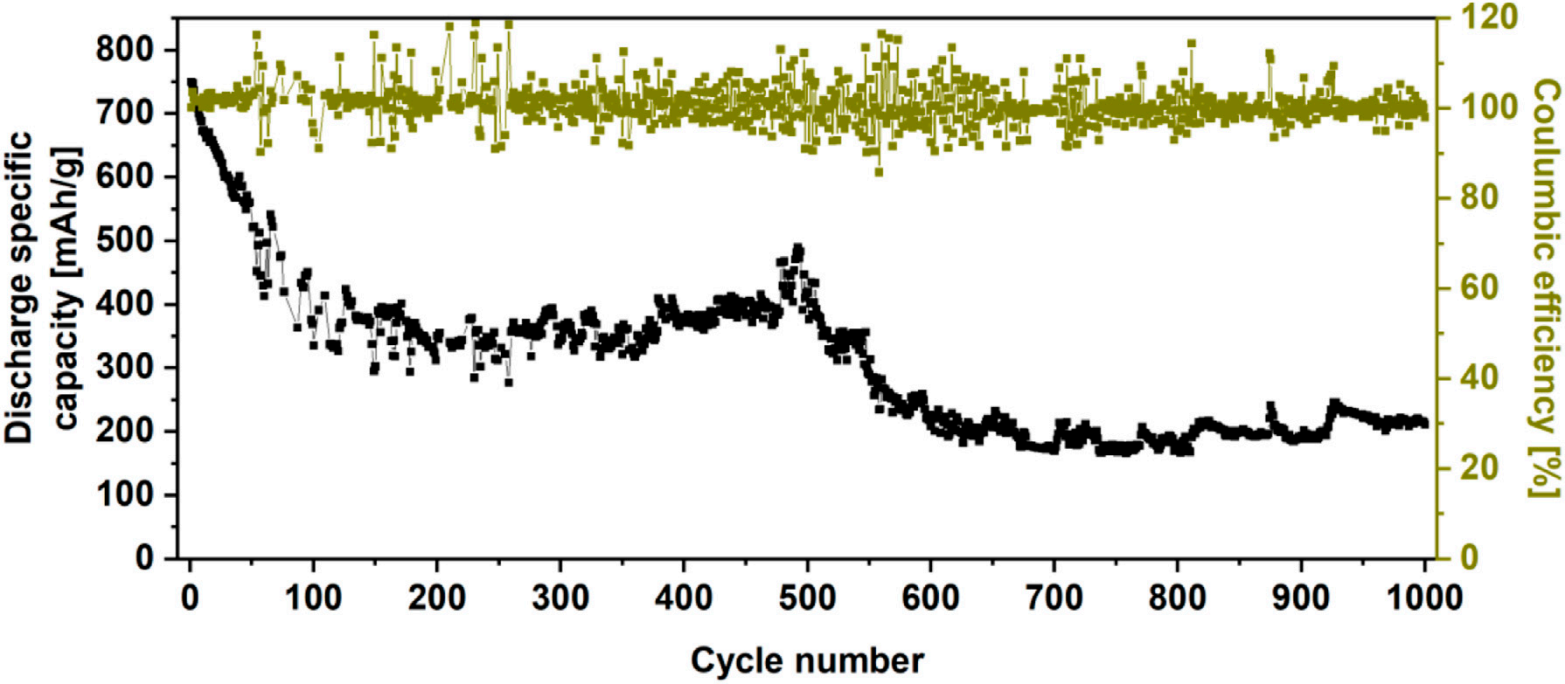
Cyclic performance of WS_2_/userGO1 recorded at 0.1 A/g.

Additionally, the WS_2_/userGO1 film was subjected to a long-term stability test (1,000 cycles, current density of 0.1 A/g) (Figure 8). The capacity faded for the first 130 cycles and stabilized at 51% of the initial capacity. After 500 stable cycles, the capacity started to fade again, losing 72% of the original capacity. However, the film displayed excellent Coulombic efficiency (101%), indicating that the cell generated more current than was used for charging. This may be related to structure collapse, leading to the fading capacity.

**FIGURE 9:**
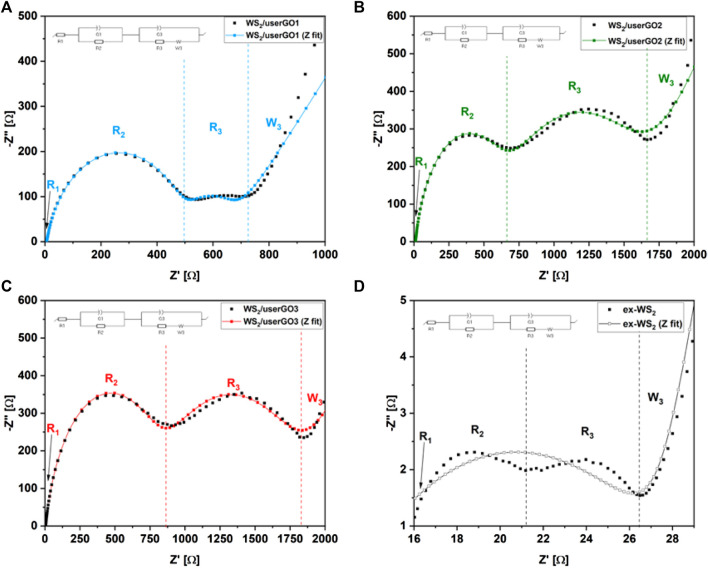
Nyquist plots of **(A)** WS_2_/userGO1, **(B)** WS_2_/userGO2, **(C)** WS_2_/userGO3, and **(D)** ex-WS_2_ after discharge/charge cycles and the equivalent circuit diagrams of the half-cells.

Figure 9 presents the Nyquist plots of the WS_2_/userGO-based films and the ex-WS_2_ sample obtained using the EIS technique. All electrodes possessed two semicircles and one slope. The first semicircle (at high frequencies range) correlated with SEI layer formation. The second semicircle (at the middle-frequencies range) corresponded to Li^+^ adsorption and charge transfer on the active material surface. It also indicated adequate device resistance. The steep slope, or Warburg impedance, confirmed the smooth Li^+^ diffusion throughout the electrodes. The values of each interphase resistance are shown in Table 2. Here, three resistances can be observed: R_1,_ or R_b_—bulk solution resistance (e.g., electrolyte, separator), R_2_, or R_ct_—charge transfer resistance, composed of ionic and electronic resistances, and R_3_, or R_int_—resistance within the cell (Lei et al., 2013). Table 2 and Figure 9 demonstrate that WS_2_/userGO-based films possess much higher resistance compared to the reference ex-WS_2_. This occurs due to two reasons: i) the USER reaction expanded the GO structure, leaving many empty cavities, thus reducing the conductivity of the graphene sheets; and ii) the ex-WS_2_ electrode was composed of 45 wt% of carbon black, which significantly increased the conductivity. However, the empty spaces of userGO are critical for the higher stability and accelerated performance of the composite materials.

**TABLE 2:** Fitted results of the equivalent circuit models in Figure 9.

Electrode	R_1_ [Ω]	R_2_ [Ω]	R_3_ [Ω]
WS_2_/userGO1	5.9	496	710
WS_2_/userGO2	10.5	630	1,650
WS_2_/userGO3	4.7	900	1940
ex-WS_2_	16.1	21	26

Table 3 compares the results presented so far in current state-of-the-art anode materials based on WS_2_ structure with respect to this work. Only the present work considers light free-standing structure based on pristine reduced graphene oxide and exfoliated WS_2_ without any non-active materials. Its electrochemical performance is among the most promising anode materials in this field.

**TABLE 3:** Comparison of different WS_2_-based structures and their performance in LIBs.

Working electrode	Structure	Discharge capacity	Ref
WS_2_/userGO1	Thin film	750 mAh/g at 0.1 A/g	This work
WS_2_/rGO	Microstructure	758.4 mAh/g at 0.1 A/g	Zhou et al. (2016b)
N-C/WS_2_	Nanosheets	635 mAh/g at 0.1 A/g	Zhao et al. (2021)
WS_2_ Nano-HS	Hollow carbon sphere	844 mAh/g at 0.1 A/g	Liu et al. (2020)
WS_2_@Gs	Onion-like nanoparticles on graphene sheets	559.5 mAh/g at 0.1 A/g	Kim et al. (2019)
WS_2_/rGO	Seaweed-like structure	720 mAh/g at 0.1 A/g	Huang et al. (2019)
NG@WS_2_@H2-rGO	N-doped graphene-coated WS_2_ nanosheets on graphene hollow spheres	593.6 mAh/g at 0.1 A/g	Li et al. (2018)

## Conclusion

This study applied a facile method to fabricate WS_2_/userGO film using a vacuum-assisted filtration technique. The synthesized three-dimensional composite film with sandwich-type architecture was treated *via* ultra-quick exothermal expansion and reduction reaction which allowed the rapid release of internally generated gases to create highly porous cavities inside the film. Additionally, this reaction improved ion transport in LIBs to promote the electrochemical performance of both film components. WS_2_/userGO films show excellent stability and electrochemical reversibility over a wide voltage range. However, the optimal electrode composed of WS_2_:userGO at a 1:1 ratio was the most electrochemically durable, with high capacity at high current densities among all samples. Furthermore, we believe that this strategy provides a universal ultra-fast route to fabricate free-standing electrodes with low mass and non-active free agents to promote both stability and capacity in energy devices.

## Data Availability

The raw data supporting the conclusion of this article will be made available by the authors without undue reservation.
