# Supplementary material for: Sandwich-type architecture film based on WS2 and ultrafast self-expanded and reduced graphene oxide in a Li-ion battery
Source: Front Chem. 2023 Jan 16;10:1102207. doi: 10.3389/fchem.2022.1102207 (PMC9885118; doi:10.3389/fchem.2022.1102207)
Supplement: Supplementary file 1 [file DataSheet1.PDF]

Supporting Information

Sandwich-type architecture film based on WS_2 and ultrafast self-expanded and reduced graphene oxide in Li-ion battery

Karolina Wenelska[1]*, Tomasz Kędzierski[1], Damian Bęben[2, 3], Ewa Mijowska[1]

[1]West Pomeranian University of Technology, Szczecin Faculty of Chemical Technology and Engineering, Department of Nanomaterials Physicochemistry, Piastow Ave. 42, 71 065 Szczecin, Poland

[2]Nanores Sp. z o.o. Sp.k., Bierutowska 57-59, 51-317 Wroclaw, Poland;

[3]Institute of Low Temperature and Structure Research, Polish Academy of Sciences in Wroclaw, Okolna 2, 50-420 Wroclaw, Poland

* Correspondence: kwenelska@zut.edu.pl

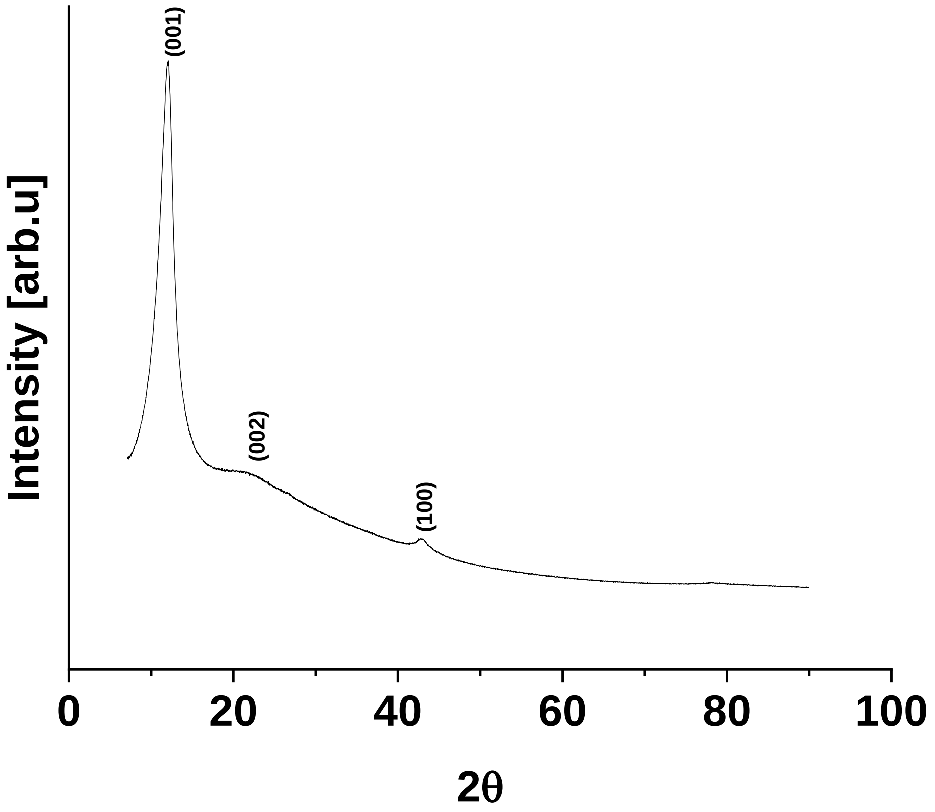

Figure S1. XRD data of GO.
